# Detrusor Smooth Muscle K_V_7 Channels: Emerging New Regulators of Urinary Bladder Function

**DOI:** 10.3389/fphys.2020.01004

**Published:** 2020-09-16

**Authors:** John Malysz, Georgi V. Petkov

**Affiliations:** ^1^Department of Pharmaceutical Sciences, College of Pharmacy, University of Tennessee Health Science Center, Memphis, TN, United States; ^2^Department of Pharmacology, College of Medicine, University of Tennessee Health Science Center, Memphis, TN, United States; ^3^Department of Urology, College of Medicine, University of Tennessee Health Science Center, Memphis, TN, United States

**Keywords:** KCNQ, smooth muscle, detrusor, excitability, contractility, overactive bladder, patch-clamp, electrophysiology

## Abstract

Relaxation and contraction of the urinary bladder smooth muscle, also known as the detrusor smooth muscle (DSM), facilitate the micturition cycle. DSM contractility depends on cell excitability, which is established by the synchronized activity of multiple diverse ion channels. K^+^ channels, the largest family of channels, control DSM excitability by maintaining the resting membrane potential and shaping the action potentials that cause the phasic contractions. Among the members of the voltage-gated K^+^ (K_V_) channel superfamily, K_V_ type 7 (K_V_7) channels — K_V_7.1–K_V_7.5 members encoded by *KCNQ1–KCNQ5* genes — have been recently identified as functional regulators in various cell types including vascular, cardiac, and neuronal cells. Their regulatory roles in DSM, however, are just now emerging and remain to be elucidated. To address this gap, our research group has initiated the systematic investigation of human DSM K_V_7 channels in collaboration with clinical urologists. In this comprehensive review, we summarize the current understanding of DSM Kv7 channels and highlight recent discoveries in the field. We describe K_V_7 channel expression profiles at the mRNA and protein levels, and further elaborate on functional effects of K_V_7 channel selective modulators on DSM excitability, contractility, and intracellular Ca^2+^ dynamics in animal species along with *in vivo* studies and the limited data on human DSM. Within each topic, we highlight the main observations, current gaps in knowledge, and most pressing questions and concepts in need of resolution. We emphasize the lack of systematic studies on human DSM K_V_7 channels that are now actively ongoing in our laboratory.

## Introduction

The urinary bladder smooth muscle, also referred to as the detrusor smooth muscle (DSM) forms the bladder wall and ultimately determines the two fundamental functions of the organ: urine storage and voiding (Andersson and Arner, 2004; Andersson and Wein, 2004). DSM relaxation along with the closure of the urinary bladder sphincter facilitates urine storage. On the other hand, coordinated DSM contractions and the opening of the urinary bladder sphincter promote urine voiding. The understanding of the complex factors regulating urinary bladder function is continuously evolving and involves myogenic, neuronal, and urothelial interacting mechanisms (see reviews by Andersson and Arner, 2004; de Groat et al., 2015; Fry and McCloskey, 2019; Dalghi et al., 2020). The myogenic concept stresses the intrinsic role of DSM cell excitability for regulating contractility. DSM cells express various types of ion channels including Ca^2+^, K^+^, non-selective cation, and Cl^–^ channels (for a general overview, please see the most recent review by Malysz and Petkov, 2020). In general, the opening of K^+^ channels causes membrane hyperpolarization, reduction of L-type voltage-gated Ca^2+^ (Ca_V_) channel open probability, decrease in net Ca^2+^ influx, and smooth muscle relaxation (Petkov, 2009; Brading and Brain, 2011; Petkov, 2012; Fry and McCloskey, 2019). Inhibition of K^+^ channels has an opposite effect, promoting DSM excitability, and thus contractility (Petkov, 2009, 2012; Brading and Brain, 2011; Fry and McCloskey, 2019; Malysz and Petkov, 2020). Among the 40 genes encoding all of the known K_V_ channels, the functions the K_V_7 subfamily are just beginning to be unraveled in the DSM of human and animal species. The K_V_7 channel subfamily contains five members — named K_V_7.1- K_V_7.5 and encoded by *KCNQ1–KCNQ5* genes. They can form either a homotetrameric (e.g., K_V_7.1) or a heterotetrameric (e.g., K_V_7.2/K_V_7.3) channel combination, with each exhibiting distinct electrophysiological and pharmacological properties (Barrese et al., 2018b). Further, K_V_7 tetrameric channel complexes may preferentially associate with regulatory accessory subunits such as KCNE that fine-tune channel biophysical properties (e.g., K_V_7.1-KCNE1) (Barrese et al., 2018b).

Recent and current medicinal chemistry efforts have discovered a number of excellent pharmacological tool compounds, either inhibitors or activators, which are K_V_7 subtype-specific. They include (1) retigabine and flupirtine, both pan-specific activators of K_V_7.2-K_V_7.5 channels; (2) ICA-069673, a selective activator of K_V_7.2/K_V_7.3 channels; (3) ML213, a preferential activator of K_V_7.2, K_V_7.2/K_V_7.3, and K_V_7.4 channels; (4) XE991 and linopirdine, pan-selective inhibitors of K_V_7.1-K_V_7.5 channels (Miceli et al., 2008, 2018; Yu et al., 2010, 2011; Amato et al., 2011; Barrese et al., 2018b). More recently, the next generation of subtype-specific and selective modulators for K_V_7.2/K_V_7.3 and K_V_7.4/K_V_7.5 channels has been described (Liu et al., 2019; Osuma et al., 2019; Zhang et al., 2019; Ostacolo et al., 2020). These compounds provide an excellent opportunity to determine the functions of K_V_7 channels subtypes in DSM and elsewhere.

Current knowledge of DSM K_V_7 channels lags behind that of other cell types where K_V_7 channels are already well recognized as critical regulators of cell function. A number of prior reviews describe in detail K_V_7 channels in smooth muscle and other cell types including regulatory mechanisms under normal and pathophysiological conditions (Stott et al., 2014; Barrese et al., 2018b; Byron and Brueggemann, 2018; Nappi et al., 2020). For non-bladder smooth muscle, transcript and protein expressions of K_V_7 channels as well as functional roles (patch-clamp electrophysiology and contractility) have been revealed in arteries (cerebral, basal, mesenteric, renal, gracilis, penile, and visceral adipose), portal vein, airway, gastrointestinal tract, uterus, and corpus cavernosum (Yeung and Greenwood, 2005; Yeung et al., 2008; Jepps et al., 2009; McCallum et al., 2009; Zhong et al., 2010; Ipavec et al., 2011; Mani et al., 2011, 2013; McCallum et al., 2011; Ng et al., 2011; Brueggemann et al., 2012a, 2014, 2018; Evseev et al., 2013; Chadha et al., 2014; Jepps et al., 2016; Barrese et al., 2018a; Stott et al., 2018; Zavaritskaya et al., 2020). The expression profiles of K_V_7 channel subtypes differ substantially based on smooth muscle cell (SMC) type. In rodent and human blood vessels, evidence points to the predominant role of K_V_7.4 and K_V_7.5 channel subtypes – but not K_V_7.2 or K_V_7.3 – regulating vascular membrane potential and contractility (Mackie and Byron, 2008; Mackie et al., 2008; Zhong et al., 2010; Ng et al., 2011; Chadha et al., 2014). In airway smooth muscle, K_V_7.1 – K_V_7.5 channel subtypes demonstrate species-specific expression profiles (Brueggemann et al., 2012a; Evseev et al., 2013). In the heart, the expressed K_V_7.1 channels co-assembled with KCNE1 proteins underlie the voltage-gated delayed rectifier K^+^ channel currents that contribute to the late repolarization phase of the cardiac action potential (Barhanin et al., 1996). Neuronal heteromeric K_V_7 channels incorporating K_V_7.2, K_V_7.3, K_V_7.4, or K_V_7.5 channel subtypes are expressed in various brain regions, and they control the membrane potential and action potential pattern generation (Brown and Passmore, 2009). Of note, the pharmacological activation of heteromeric K_V_7.2/K_V_7.3 channels is thought to underlie the primary mechanism of action for retigabine, a previously approved anti-epileptic drug (Wickenden et al., 2000). Mechanistically, retigabine shifts the voltage dependency of K_V_7.2–K_V_7.5 channels to hyperpolarized membrane potentials (due to an increase in open probability), accelerates the activation, and reduces deactivation of the currents (Tatulian et al., 2001; Tatulian and Brown, 2003). A single tryptophan within the S5 domain of K_V_7.2-K_V_7.5 channels has been shown to be essential for the effect of retigabine (Schenzer et al., 2005).

This review summarizes our current understanding of the K_V_7 channels’ physiological roles in DSM. First, we highlight the initial findings for retigabine, a K_V_7.2–K_V_7.5 channel activator causing urinary retention in clinical trials for epilepsy, which suggested for the first time a role for K_V_7 channels in the control of urinary bladder function. Next, we provide an overview of subsequent *in vivo* animal model studies of urinary bladder function with retigabine and other K_V_7 channel modulators. Then, we describe the current understanding of the roles of K_V_7 channels in DSM based on systematic studies that our group — initially at the University of South Carolina and now at the recently established Urology Research Center, University of Tennessee, Memphis — has pioneered in this field and those of other investigators. We summarize the expression profiles for K_V_7 channel subtypes in DSM whole-tissue and single-cell preparations, electrophysiological findings for K_V_7 channel modulators in DSM cell patch-clamp and tissue conventional microelectrode electrophysiological experiments, and how K_V_7 channel pharmacological modulators affect intracellular Ca^2+^ concentrations and DSM contractility. Within each topic, we highlight the main findings and current knowledge gaps and emphasize the most pressing questions and concepts regarding DSM K_V_7 channels that await scientific resolution.

## K_V_7 Channel Pharmacological Activation Is Associated With Urinary Retention in Patients

The initial realization that K_V_7 channels may be involved in regulating urinary bladder function dates to the very first clinical testing of retigabine as an adjunct therapy for controlling epilepsy (Brickel et al., 2012). Safety analyses of clinical data for retigabine (phases 2/3) revealed urinary retention as a notable side-effect. Indeed, subjects taking retigabine in comparison to placebo reported a ∼2-fold higher incidence of urinary retention (0.9% versus 0.5%) and a relative risk of 1.32 (95% confidence interval, 0.986 – 1.761) of reporting a urinary/renal side effect (Brickel et al., 2012). Intriguingly, these findings suggested that retigabine, by activating K_V_7 channels, might prove beneficial in ameliorating overactive bladder/detrusor overactivity. Retigabine, however, has not been clinically examined specifically for any urinary bladder condition. In contrast, its very close structural analog flupirtine has progressed into a phase 2 study for overactive bladder (Michel et al., 2012). Flupirtine has a long history (since 1981) of clinical use in Europe (but was not approved in the US) as a centrally acting, non-opioid analgesic (Devulder, 2010). Unfortunately, discoveries of other unexpected side-effects hampered clinical uses of retigabine and flupirtine (Brickel et al., 2012; Michel et al., 2012). For retigabine, this includes a prolongation of the QT interval and potential development of cardiac arrhythmias in certain patients (Barrese et al., 2010; Splinter, 2013). The mechanism involved remains to be elucidated; however, a direct effect on the heart is unlikely. When tested in guinea pig and human cardiomyocytes, retigabine induced a reduction rather than a prolongation of the action potential (Rubi et al., 2017). Other limitations for retigabine and flupirtine are their non-selectivity among K_V_7 channel subtypes (both are active at K_V_7.2-K_V_7.5 channels), and their relatively weak potency (Miceli et al., 2008, 2011, 2018). Thus, novel subtype-specific K_V_7 channel activators are needed. Both retigabine and flupirtine remain as excellent tools for preclinical investigations of K_V_7 channels, including their roles in DSM, as described below.

## K_V_7 Channel Modulators Affect Urinary Bladder Function *in vivo* in Experimental Animal Models

A seminal report, published back in 2004, described that retigabine altered urinary bladder function *in vivo* (Streng et al., 2004). In conscious female adult rats, with continuously monitored bladder function by cystometry, retigabine applied intravenously (i.v., 0.5–5 mg/kg), intracerebroventricularly (10 or 50 μg bolus), and intravesically (100–1000 ng/ml) increased micturition volume and voiding intervals, and when given intravesically, decreased capsaicin-induced DO. Since XE991, a pan-selective K_V_7 channel blocker, completely inhibited the effects of retigabine, the study authors concluded that *“KCNQ channels can be interesting targets aiming at micturition control”* (Streng et al., 2004).

A later report on unanesthetized adult female rats confirmed the inhibitory effects of retigabine (applied orally at 0.5 mg/kg) on capsaicin-induced DO where the K_V_7 channel activator compound retigabine decreased micturition volume output (Svalo et al., 2012). This study also demonstrated retigabine efficacy in an animal model of acetic-acid-induced DO examined under anesthesia. Specifically, retigabine (i.v.) increased micturition interval (doses 0.001–1 mg/kg) and micturition volume (0.1 mg/kg). While cardiovascular effects on blood pressure were observed, they occurred only at the highest dose tested (1 mg/kg). The lower doses (0.01–0.3 mg/kg) still displayed positive effects on urinary bladder function, and these data, thus, showed separation of desirable urinary bladder effects and unwanted cardiovascular effects *in vivo*. Similarly, in rats, retigabine at a dose of 0.1 mg/kg (i.p.) reduced the frequency of spontaneous contractions during bladder filling, and it also completely abolished (10 mg/kg i.p.) acetic acid-enhanced (0.25%) micturition activity (Argentieri and Sheldon, 2006).

More recently, the effects of retigabine (0.01–3 mg/kg, i.v.) were examined on rhythmic bladder contractions (RBCs) in adult female rats under anesthesia (Aizawa et al., 2017). Retigabine dose-dependently decreased both the frequency and amplitude of RBCs with the former parameter showing higher sensitivity. This study also revealed a reduction in afferent nerve fiber firing activities of myelinated Aδ and unmyelinated C-fibers by retigabine at 1 mg/kg, but not at lower doses (Aizawa et al., 2017). Interestingly, retigabine at 0.3 mg/kg reduced the frequency of RBCs by ∼50% without affecting the afferent neuronal firing, suggesting that the primary cellular site of action for retigabine at this particular dose involved DSM (Aizawa et al., 2017).

Similarly, in adult mice, retigabine almost completely attenuated the afferent nerve firing associated with RBCs (referred to as transient contractions/TCs in this publication) during *ex vivo* bladder filling (Tykocki et al., 2019). Of note, retigabine reduced the magnitude of RBCs (i.e., TC integral in the report). Since XE991 prevented the effects of retigabine on afferent firing and RBCs, the findings supported the involvement of K_V_7 channels. Interestingly, XE991 examined alone did not change the RBC magnitude (integral), but rather its frequency, in contrast to observations with retigabine (Tykocki et al., 2019).

Collectively, K_V_7 channel modulators exhibit *in vivo* efficacy in experimental animal models, supporting that K_V_7 channels play a regulatory role in the urinary bladder. Below, we summarize the experimental evidence based on systematic studies initiated and continued today by our group and others on K_V_7 channel subtypes in DSM by describing their expression and function in DSM.

## Expression of K_V_7 Channel Subtypes in DSM Whole-Tissue and Single Cells

K_V_7 channel subtypes have been detected at mRNA or protein levels in guinea pig, rat, and human DSM whole tissues or isolated cells (Svalo et al., 2012; Afeli et al., 2013; Anderson et al., 2013; Svalo et al., 2013, 2015; Provence et al., 2015, 2018). An important consideration for interpretation of the results from whole-DSM tissue preparations is that — since RT-PCR is a very sensitive technique —a detected mRNA signal can potentially originate from any cell type present in the whole tissue preparation, including DSM cells, interstitial cells, nerve fibers, and fibroblasts. Thus, to conclude that a given mRNA product is expressed directly in DSM cells, positive detections in isolated single DSM cells are required. In adult male guinea pig DSM whole-tissues and single cells, all K_V_7.1–K_V_7.5 were detected at mRNA and protein levels (Afeli et al., 2013; Anderson et al., 2013; Provence et al., 2015). Our qRT-PCR experiments revealed the following rank order of expressions in DSM whole-tissue: (K_V_7.1∼K_V_7.2 > K_V_7.3∼K_V_7.5 > K_V_7.4) and single cells (K_V_7.1∼K_V_7.2 > K_V_7.5 > K_V_7.3∼K_V_7.4) (Afeli et al., 2013). Further confirmations were made with RT-PCR, immunohistochemistry (with co-labeled DSM cells), and DSM cell immunocytochemistry determinations (Afeli et al., 2013; Anderson et al., 2013; Provence et al., 2015). Since K_V_7 channels can assemble as preferential heteromers, the expression data suggested that guinea pig DSM K_V_7 channels could potentially comprise K_V_7.2/K_V_7.3, K_V_7.3/K_V_7.5, and K_V_7.4/K_V_7.5 heteromeric complexes as well as homomeric channels. Experimental evidence from DSM cells based on *in situ* proximity ligation assay supported the presence of K_V_7.4/K_V_7.5 channel complexes (Provence et al., 2018). The positive detection of K_V_7.2 and K_V_7.3 subtypes in DSM cells along with functional findings of efficacy on excitability and contractility by the selective K_V_7.2/K_V_7.3 channel activator ICA-069673 were consistent with the existence of K_V_7.2/K_V_7.3 heteromeric channels (Provence et al., 2015). A remaining question is to determine which of the possible homomeric or heteromeric combinations comprise the most physiologically relevant native DSM K_V_7 channel.

In contrast, the published K_V_7 channel expression data in rats, pigs, and humans are only available for whole-DSM tissues (Argentieri and Sheldon, 2006; Svalo et al., 2012, 2013, 2015; Bientinesi et al., 2017; Seefeld et al., 2018). Although initially in rat DSM mRNAs for K_V_7.1, K_V_7.3, and K_V_7.5 channels were detected, subsequent studies also confirmed the presence of K_V_7.4 channels (Argentieri and Sheldon, 2006). In adult female rats, qRT-PCR analysis showed the highest level of expression for the K_V_7.4 subtype followed by lower but still detectible K_V_7.1 and K_V_7.5 subtypes and marginal/not present K_V_7.2 and K_V_7.3 subtypes (Svalo et al., 2012). Western blot studies of whole DSM tissue preparations found protein expression for K_V_7.4 but not K_V_7.2 (Svalo et al., 2012). Comparative parallel qRT-PCR expression analyses on the heart (K_V_7.1 > K_V_7.4 > K_V_7.2∼K_V_7.3 > K_V_7.5), aorta (K_V_7.1 > K_V_7.4 > K_V_7.5 > K_V_7.3 > K_V_7.2), and brain (K_V_7.3 > K_V_7.2∼K_V_7.5 > K_V_7.4 > K_V_7.1) revealed differential mRNA expression profiles for K_V_7 channel subtypes (Svalo et al., 2012). The relative level of K_V_7.4 channel mRNA expression in the bladder approximated or reached a level slightly lower than that identified for the other three organs (Svalo et al., 2012). qRT-PCR analyses of K_V_7.3–K_V_7.5 channel mRNAs in pig DSM tissue provided similar findings with the following rank order of expression: K_V_7.4 > K_V_7.5 > K_V_7.3 subtypes (Svalo et al., 2013). For the K_V_7.4 subtype, the relative levels of mRNA expression were 0.6-fold and 2.8-fold lower in the bladder than in the cortex and the heart (Svalo et al., 2013). When compared to K_V_7.3 and K_V_7.5 channel subtypes, the relative DSM mRNA expression levels of the K_V_7.4 channel subtype were, respectively, 10- and 2.5-fold lower in the heart and 200- and 2500-fold lower in the cortex (Svalo et al., 2013). These observations reinforced a high level of expression for K_V_7.4 channels in DSM and organ-specific expression profiles of K_V_7 channel subtypes.

Initial qRT-PCR investigations on human whole DSM tissue preparations detected K_V_7 channel subunits with relative mRNA expression: K_V_7.3∼K_V_7.4∼K_V_7.5∼K_V_7.1 > K_V_7.2 (Svalo et al., 2015). Two other groups found a similar rank order in human DSM tissues: K_V_7.4 ≥ K_V_7.5∼K_V_7.1 > K_V_7.3 > K_V_7.2 (Bientinesi et al., 2017) and K_V_7.4∼K_V_7.5 > K_V_7.3 > K_V_7.2 (Seefeld et al., 2018). Thus, these studies identified the K_V_7.4 channel subtype as the most highly expressed while the K_V_7.2 channel subtype was the lowest. There is, however, an earlier publication where mRNAs for only K_V_7.3 and K_V_7.5 channels were detected but not for K_V_7.1, K_V_7.2, and K_V_7.4 channels in human bladder specimens, which raised the possibility of some experimental variability (Argentieri and Sheldon, 2006). Interestingly, in DSM tissues obtained from urinary bladders with partial outlet obstruction (POO) due to benign prostatic hyperplasia (BPH), the transcript expression of K_V_7.1 channels increased 3.4-fold (Svalo et al., 2015). For other K_V_7 subtypes, mRNA expression remained unchanged except for K_V_7.2 channels, which were undetectable in POO-bladders (Svalo et al., 2015). The observed K_V_7.1 channel expression increase may reflect a compensatory upregulation that developed to counteract DO under POO. Since accessory KCNE subunits can assemble with K_V_7 channels altering the K_V_7 channel complex properties, it is of interest to elucidate their expression and function in DSM. Currently, only a single report has provided such expression data using qRT-PCR. That analysis showed transcript expressions of all KCNE1-5 subunits with comparative relative mRNA expressions, which did not change under POO due to BPH (Svalo et al., 2015). A key limitation of human and animal model expression studies is the lack of data on single DSM cells at both mRNA and protein levels. Thus, future studies of DSM specimens from patient-donors and animal models with both normal and aberrant bladder function are needed.

## K_V_7 Channel Functional Studies on DSM Cell Excitability

### Electrophysiological Characterizations of K_V_7 Channels in Regulating DSM Cell Excitability

#### Studies on Single DSM Cell Excitability

**FIGURE 1:**
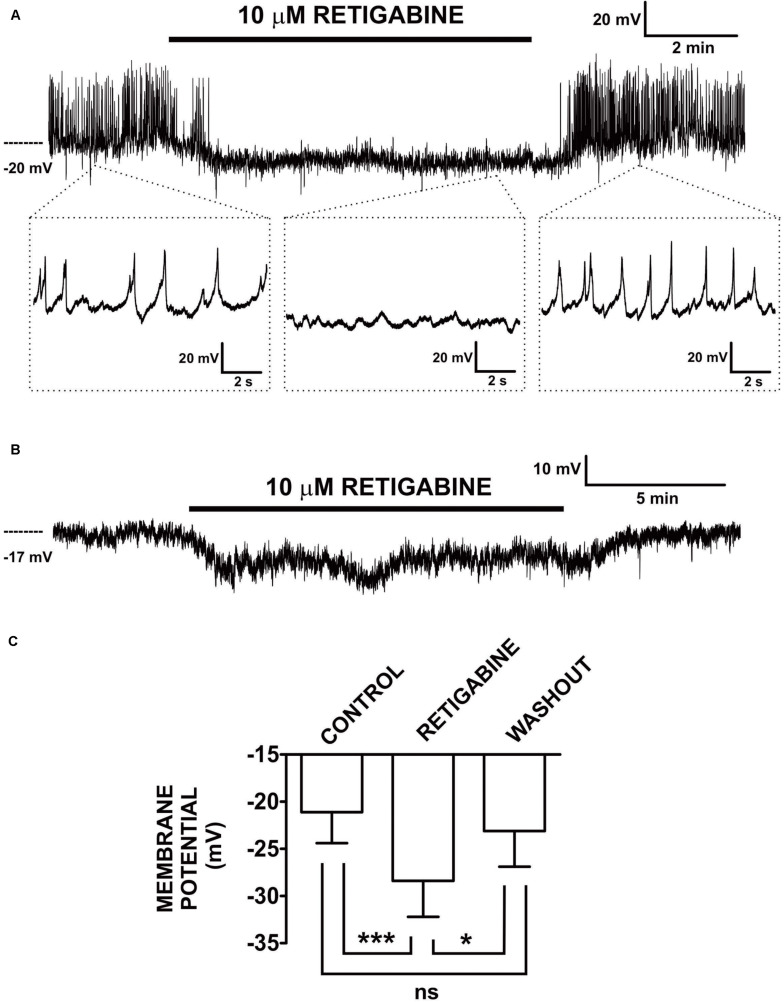
Retigabine, a K_V_7.2–K_V_7.5 channel activator, induced hyperpolarization and inhibited generation of spontaneous action potentials in freshly isolated guinea pig DSM single cells. **(A)** An original current-clamp membrane potential recording in a DSM cell — obtained with the amphotericin-B perforated patch-clamp method — shows spontaneous action potentials. Retigabine (10 μM) inhibited these spontaneous action potentials and caused membrane hyperpolarization. Upon washout, membrane potential and action potentials fully recovered. The insets in **(A)** depict electrical activity on an expanded time scale for the time points indicated. **(B)** An original membrane potential recording from a DSM cell in which spontaneous action potentials were absent. Retigabine (10 μM) induced membrane hyperpolarization that recovered following washout of the compound. **(C)** Summary data show statistically significant hyperpolarization of DSM cells by retigabine (10 μM) (*n* = 12 DSM cells, *N* = 11 guinea pigs) and the recovery from hyperpolarization upon its washout (*n* = 8 DSM cells, *N* = 7 guinea pigs). The bars depict the actual mean membrane potential and SEM values for each experimental condition. The comparisons showed statistical significance of ***P < 0.001 and *P < 0.05 for the specified conditions, ns, non-significant (*P* > 0.05). This figure is a reproduction from our previous *PLoS On*e publication (Afeli et al., 2013) and used according to the publisher’s copyright policy.

The initial evidence for a role of K_V_7 channels in determining DSM cell excitability dates back to 2013. At that time, two independent research laboratories, Karen McCloskey’s and our group, found that retigabine and flupirtine, both K_V_7.2–K_V_7.5 channel activators, affected electrophysiological properties of guinea pig DSM cells (Afeli et al., 2013; Anderson et al., 2013). As shown in Figure 1, retigabine hyperpolarized the DSM cell membrane potential by ∼7 mV when examined with the perforated whole-cell patch-clamp technique (Afeli et al., 2013). Of note, in DSM cells that exhibited spontaneous action potentials, retigabine caused their inhibition via membrane potential hyperpolarization (Afeli et al., 2013). This key finding, thus, mechanistically linked activation of K_V_7 channels with inhibition of L-type Ca_V_ channels in DSM cells. In a separate study using the conventional whole-cell approach, voltage step-induced K^+^ currents were increased by flupirtine and also meclofenamic acid, another K_V_7 channel activator displaying preference for K_V_7.2/K_V_7.3 channels (Anderson et al., 2013). Flupirtine also caused a reversible hyperpolarization of ∼5 mV in DSM cells measured with the conventional whole-cell current-clamp method (Anderson et al., 2013). XE991 and linopirdine, both K_V_7.1–K_V_7.5 channel blockers, displayed effects opposite of those of either retigabine or flupirtine (Anderson et al., 2013; Provence et al., 2015, 2018). XE991 induced DSM cell depolarization (examined with either the perforated or conventional whole-cell current-clamp approach) and inhibited the voltage-step-induced K^+^ currents (conventional whole-cell) (Anderson et al., 2013; Provence et al., 2015, 2018). Linopirdine mimicked the effects of XE991 on the membrane potential (conventional current-clamp) and whole-cell K^+^ currents (conventional voltage-clamp). Additionally, chromanol 293B, a K_V_7.1 and K_V_7.1/KCNE1 channel inhibitor (Lerche et al., 2000), attenuated the voltage-step induced K^+^ currents (conventional whole-cell) in DSM cells suggesting a regulatory role of this channel subtype (Anderson et al., 2013). Collectively, these electrophysiological studies solidified the notion that multiple K_V_7 channel subtypes determine guinea pig DSM cell excitability.

Recent medicinal chemistry efforts have led to the discovery of new channel-selective tool compounds for the study of K_V_7 channel subtypes. These novel compounds exhibit K_V_7 channel subtype preferential targeted profiles. Two such novel K_V_7 channel activators are ICA-069673 and ML213 (Yu et al., 2010, 2011; Amato et al., 2011). ICA-069673 has 20-fold or higher selectivity for K_V_7.2/K_V_7.3 channels (EC_50_ = 0.69 μM) over K_V_7.3/K_V_7.5 and K_V_7.1 channels, and this novel K_V_7 activator exhibited no measurable activity against a panel of cardiac ion channels including hERG, Na_V_1.5, L-type Ca_V_ and K_V_7.1 channels (Amato et al., 2011). ML213 shows a preference for K_V_7.2 (EC_50_ = 0.33 μM), K_V_7.2/K_V_7.3 (EC_50_ = 0.37 μM), and K_V_7.4 (EC_50_ = 0.51 μM) channels with a high degree of selectivity (at least 80-fold, EC_50_ > 30 μM) over K_V_7.1, K_V_7.1/KCNE1, K_V_7.3, and K_V_7.5 channels (Yu et al., 2010, 2011). Both ICA-069673 and ML213, when studied on guinea pig DSM isolated cells with the perforated whole-cell patch-clamp approach, hyperpolarized the cell membrane potential in an XE991-dependent manner (Provence et al., 2015, 2018). Specifically, pretreatment of DSM cells with XE991 prevented the hyperpolarizing effects of ICA-069673 and ML213 (Provence et al., 2015, 2018). Further, XE991 applied in the presence of either one of these K_V_7 channel activators caused reversal of the compound-induced membrane hyperpolarization (Provence et al., 2015, 2018).

The pharmacological efficacy of ML213 extends to DSM whole-cell K^+^ currents (Provence et al., 2018). ML213 increased voltage-step induced K^+^ currents under experimental conditions that maximized the detection of K_V_7 channel currents, specifically recording the electrical activity in the presence of paxilline, a BK channel inhibitor, and GdCl_3_, an inhibitor of both L-type Ca_V_ and nonselective cation channels, using a depolarized holding potential of -10 mV, which ensures inactivation of non-K_V_7 channels (Provence et al., 2018). The ML213-induced channel activation was fully reversible upon washout of the compound (Provence et al., 2018). Indeed, this seminal finding made by our group was the very first-ever successful recording of native K_V_7 channel currents in DSM cells with the perforated patch-clamp approach, complementing previous findings with the conventional whole-cell approach (Anderson et al., 2013; Provence et al., 2018). The studies with ICA-069673 and ML213, therefore, demonstrated that K_V_7 channel subtype-specific targeting by selective pharmacological activation decreases DSM cell excitability by promoting hyperpolarization.

In contrast to results for these animal models, the precise physiological role of K_V_7 channels in human DSM cells has yet to be evaluated by systematic approaches. Initial data from our group revealed the effects of K_V_7 channel modulators on single DSM cell excitability (Provence et al., 2015, 2018). In these studies, single human DSM cells were obtained using a highly optimized methodology (Malysz et al., 2020). Retigabine and XE991, respectively, hyperpolarized and depolarized DSM cell membrane potentials recorded with the amphotericin perforated current-clamp (Provence et al., 2015, 2018). Retigabine also increased ramp-protocol evoked K_V_ currents under experimental conditions optimized for recording of native K_V_7 currents with the perforated voltage-clamp approach (Provence et al., 2018). In addition, a preliminary report on cultured human DSM cells using patch-clamp electrophysiology revealed retigabine induced membrane potential hyperpolarization and an increase in voltage-step-induced currents. Future comprehensive studies — already underway in our laboratory — will reveal the specific details as to how K_V_7 channel modulators, both pan- and subtype-selective, alter human DSM cell excitability.

Two other common animal models, rat and mouse, have not been comprehensively evaluated for the electrophysiological properties of DSM K_V_7 channels. A patent application from Wyeth/Pfizer (Argentieri and Sheldon, 2006), revealed that in rat DSM cells, retigabine (1) enhanced voltage step- or ramp-induced K^+^ currents by 2–3-fold as measured, and (2) hyperpolarized the membrane potential by ∼10 mV (Argentieri and Sheldon, 2006). Subsequent applications of XE991 in the presence of retigabine at least partially reversed the effects of retigabine on whole-cell currents and the cell membrane potential in rat DSM cells (Argentieri and Sheldon, 2006).

The role of K_V_7 channels in mouse DSM cells has not been well studied. Currently, there are no publications using direct molecular biology approaches to detect K_V_7 channel expression in mouse DSM, and the single available report using patch-clamp electrophysiology did not record K_V_7 currents in mouse DSM cells, most likely due to the use of non-optimal conventional whole-cell patch-clamp recording protocols (Tykocki et al., 2019). The utilized protocol consisted of 500 ms duration depolarizing steps from −60 to +20 mV (10 mV increments with a 10 s pause between steps), but the holding potential was not specified although it appeared to be ≤−60 mV (Tykocki et al., 2019). The voltage-step induced K^+^ currents that they recorded were reduced by XE991 (10 μM, at +20 mV from ∼ 6 to 3 pA/pF) but not affected by retigabine, although kinetic response time courses and washout effects were not reported (Tykocki et al., 2019). A possible confounding issue related to the interpretation of the pharmacological effects of XE991 in that study was that this compound also inhibits K_V_2.1 and K_V_2.1/K_V_9.3 channel currents at 10–100 μM rather modestly (20–25%); in comparison, IC_50_ values of XE991 (0.6–5.5 μM) for K_V_7 channel subtypes are much lower (Wang et al., 1998; Wladyka and Kunze, 2006; Zhong et al., 2010). K_V_2.1 channels and currents have been reported in mouse DSM cells (Thorneloe and Nelson, 2003) and also in guinea pig and likely in human DSM cells (Hristov et al., 2012a,b). Of note, retigabine (0.3–3 μM) is known to effectively inhibit recombinant K_V_2.1 channel currents (Stas et al., 2016) and, therefore, is expected to attenuate K_V_2.1 channel currents in DSM cells. Furthermore, native SMC K_V_7 channel currents are best studied using an optimized voltage protocol and the perforated patch-clamp method (Mani et al., 2011, 2013, 2016; Brueggemann et al., 2012b, 2014, 2018), which was not used in the Tykocki et al. (2019) study. The optimized K_V_7 current recording protocol involves holding SMCs at a very depolarized membrane potential such as –4 mV and conducting voltage steps within a range of voltages from −70 mV up to ∼+20 mV for at least 1 s (1–5 s) and, importantly, in the presence of Gd^3+^. Gd^3+^ blocks non-selective cation currents as well as L- and T-type Ca_V_ channels, while the highly depolarized membrane potential promotes inactivation of non-K_V_7 channel currents (Mani et al., 2011). These specific conditions facilitate experimental isolation of native K_V_7 channel currents. Indeed, native K_V_7 channel currents in guinea pig DSM cells were previously recorded using this optimized protocol and in the presence of the BK channel blocker paxilline. These K_V_7 currents were increased by the novel K_V_7 channel activator ML213 (Provence et al., 2018). Tykocki et al. (2019) also reported that DSM cell membrane potentials (∼−45 mV on average and the majority of DSM cells ≤ −40 mV, up to ∼−60 mV), recorded with the perforated patch-clamp approach, were not hyperpolarized by retigabine (Tykocki et al., 2019). Not surprisingly, whole-cell currents (conventional patch-clamp method) measured at −40 mV in a gap-free recording mode under elevated extracellular K^+^ (*E*_K_ = −10 mM) were not affected by 10 μM retigabine (Tykocki et al., 2019). In contrast, in guinea pig DSM cells where the DSM cell membrane potential is more depolarized at ∼−20 mV (perforated patch-clamp method), retigabine induced hyperpolarization (Afeli et al., 2013). Hence, the dissimilar effects of retigabine reported by Tykocki et al. (2019) may be related to specific experimental conditions, including the membrane potential level and voltage-protocol steps utilized, or to species differences. Tykocki et al. (2019) provided a caveat regarding mouse urinary bladder smooth muscle (UBSM) *“it is conceivable that K_V_7 currents could be present and necessary for maintaining UBSM, but were undetectable using our patch-clamp parameters”* (Tykocki et al., 2019). Hence, future studies should address what may have been methodological issues and confirm the functional presence of K_V_7 channels in mouse DSM cells using more precise patch-clamp protocols for recording native K_V_7 channel currents.

Tykocki et al. (2019) also suggested that retigabine may cause partial inhibition of the L-type Ca_V_ channels in mouse DSM cells, consistent with prior findings (Mani et al., 2013; Rubi et al., 2017). At 10 μM, retigabine weakly attenuated the mouse DSM inward voltage-gated Ca_V_ current by ∼25% (from 8 to 6 pA/pF, evoked by the voltage step from −60 to +20 mV), while 60 mM KCl induced tonic contraction (primarily mediated via L-type Ca_V_ channel activation) by ∼20% (Tykocki et al., 2019). Similarly, recombinant peak L-type Ca_V_ channel currents (human Ca_V_1.2 channels expressed in ts201 cells) were reduced ∼10 and ∼50% by 10 and 100 μM retigabine, respectively, while native L-type Ca_V_ currents in basal artery SMCs displayed a slightly higher sensitivity to 10 μM retigabine and showed ∼60% inhibition in the peak Ca_V_ channel current amplitude at +10 mV (Mani et al., 2013). In contrast to retigabine, the dihydropyridine L-type Ca_V_ channel blocker isradipine almost completely inhibited both the mouse DSM inward voltage step-induced Ca_V_ channel currents (from ∼−3.5 to −0.5 pA/pF, evoked by the step from −80 to +0 mV) and the 85 mM K^+^ induced tonic contraction by >85% (Wegener et al., 2004). Similarly, in guinea pig DSM cells and DSM strips, nifedipine induced comparable near-complete inhibition of inward L-type Ca_V_ channel currents and 60 mM K^+^ induced tonic contractions (Malysz et al., 2014; Provence et al., 2015). However, since comparative concentration-responses to retigabine attenuating DSM strip contractility under low (physiological; e.g., 5 or 20 mM) and elevated (e.g., 60 mM) extracellular K^+^ were not yet studied in mouse DSM including by Tykocki et al. (2019) it remains unknown how the relatively minor inhibition on L-type Ca_V_ channels contributes to the overall effect of retigabine in mouse DSM. Hence, the conclusion that L-type Ca_V_ channel blockade accounts solely for the effect of retigabine on mouse DSM requires validation and additional experimental evidence. In contrast, elevation of extracellular K^+^ from 5 or 20 to 60 mM reduced retigabine-, ICA-069673-, and ML213-induced relaxation in rat or guinea pig DSM strips, consistent with a K^+^ conductance underlying the effect of these K_V_7 channel activators on DSM contractility (Argentieri and Sheldon, 2006; Rode et al., 2010; Provence et al., 2015). Given the potential confounding issues with retigabine and XE991 — especially their selectivity — systematic examinations of the effects of newer K_V_7 channel modulators on DSM cells should reveal what role K_V_7 channels play in the regulation of mouse DSM excitability. Such additional studies should also involve determinations of expression profiles of K_V_7 channel subtypes in freshly isolated DSM cells (mRNA and protein), whole tissues, and afferent urinary bladder neurons, as these datasets are currently not well understood. Indeed, published findings by multiple groups have confirmed expressions and functional roles of K_V_7 channels in guinea pig, rat, pig, and human DSM tissues and/or isolated DSM cells (Argentieri and Sheldon, 2006; Rode et al., 2010; Svalo et al., 2012, 2013, 2015; Afeli et al., 2013; Anderson et al., 2013; Provence et al., 2015, 2018; Bientinesi et al., 2017).

#### Electrophysiological Investigations of DSM Muscle Bundle/Tissue Strip Preparations

Pharmacological effects of K_V_7 channel modulators have been examined with the conventional (“sharp”) microelectrode electrophysiology in DSM muscle bundles/strips and muscularis mucosae smooth muscle (MMSM) preparations in the guinea pig urinary bladder. These two types of urinary bladder smooth muscle display similar types of electrical activity encompassing electrical quiescence as well as regular and irregular action potentials (Takagi and Hashitani, 2016; Lee et al., 2018). Flupirtine either completely abolished DSM action potentials or slowed their generation by decreasing frequency without any changes in the resting membrane potential attenuated or in action potential parameters (Takagi and Hashitani, 2016). On the other hand, XE991 induced membrane potential depolarization (∼4 mV) and this effect was associated with an increased action potential discharge (Takagi and Hashitani, 2016). In MMSM intact preparations, XE991 alone did not alter any of the action potential parameters or the resting membrane potential, although an increase of ∼30% in the number of spontaneous action potentials occurring during a burst of action potentials was observed (Lee et al., 2018). Flupirtine decreased the MMSM peak action potential amplitude by ∼25%, and the action potential after-hyperpolarization amplitude by ∼15%, without a significant change in resting membrane potential (Lee et al., 2018). These observations suggest a relatively higher contribution of K_V_7 channels to the regulation of DSM excitability rather than of MMSM in guinea pigs, although in both types of urinary bladder smooth muscle, pharmacological modulators of K_V_7 channels clearly affected the function.

In summary, evidence supports a role for K_V_7 channels in regulating DSM excitability, but very few studies are currently available, thus additional systematic research efforts are warranted, especially on human DSM, and such systematic studies are currently underway in our laboratory.

### K_V_7 Channel Pharmacological Modulators Affect DSM Contractility and Intracellular Ca^2+^

#### Studies of DSM Contractility in Experimental Animal Models

As of now, the guinea pig is the most thoroughly characterized species for determining the functional effects of K_V_7 channel modulators, both activators and blockers, on DSM contractility. Our group and others have evaluated how diverse compounds acting on K_V_7 channels affect spontaneous phasic, 20 mM KCl-induced, and electrical field stimulated (EFS)-induced contractions (Tables 1, 2). K_V_7 channel activators — including retigabine (pan-selective for K_V_7.2–K_V_7.5 channels), flupirtine (pan-selective for K_V_7.2–K_V_7.5 channels), L-364373 (active on K_V_7.1 channels), ICA-069673 (selective for K_V_7.2/K_V_7.3 channels), ML213 (preferential selectivity for K_V_7.2, K_V_7.2/K_V_7.3, and K_V_7.4 channels), and meclofenamic acid (preferential activity on K_V_7.2/K_V_7.3 channels)—have shown inhibitory effects on guinea pig DSM contractions as summarized in Table 1. Conversely, K_V_7 channel blockers — XE991 and linopirdine (both inhibitors of K_V_7.1–K_V_7.5 channels) and chromanol 293B (a K_V_7.1 channel inhibitor) — enhanced DSM contractility as summarized in Table 2. Of critical importance are the findings of pretreatment of DSM tissue strips with an inhibitor such as XE991 on subsequent relaxation-inducing responses of K_V_7 channel activators. Since K_V_7 channel inhibition attenuated or prevented the subsequent effects of K_V_7 channel activators, these findings substantiated that the underling pharmacological mechanism-of-action for the compounds involves K_V_7 channels. Specifically, XE991 effectively attenuated both ICA-069673-induced and ML213-mediated inhibitions of DSM spontaneous phasic contractions (Provence et al., 2015, 2018). Collectively, to date all published pharmacological studies using diverse K_V_7 channel modulators support a role of multiple K_V_7 channel subtypes in the regulation of guinea pig DSM contractility.

**TABLE 1:** Effects of K_V_7 channel activators on DSM contractility in various animal species and humans.

**Activator**	**Species**	**Experimental DSM contraction protocol**	**Observation on DSM contractility**	**References**
L-364373 (K_V_7.1 channel activator)	Guinea pig	Spontaneous phasic	Inhibition of phasic contractions, effects on amplitude and muscle force, but weak changes in frequency and duration	Afeli et al., 2013
	Guinea pig	EFS-induced	Inhibition of EFS contractions, effects on amplitude and muscle force, but a weak change in the duration	Afeli et al., 2013

Retigabine (K_V_7.2–K_V_7.5 channel activator)	Guinea pig	Spontaneous phasic	Inhibition of phasic contractions, effects on amplitude, muscle force, frequency, and duration	Afeli et al., 2013
	Rat	Spontaneous phasic	Inhibition of phasic contractions, effects on amplitude and tension	Rode et al., 2010; Wang et al., 2014
	Rat	Carbachol-induced phasic	Inhibition of phasic contractions, effects on amplitude and tension	Rode et al., 2010
	Pig	Carbachol-induced phasic	Inhibition of phasic contractions, effects on amplitude and tension	Svalo et al., 2013
	Human	Carbachol-induced tonic	Inhibition of tonic contraction	Svalo et al., 2015
	Human	Bethanechol-induced tonic	Inhibition of tonic contraction	Bientinesi et al., 2017
	Rat	20 mM KCl-induced phasic	Inhibition of phasic contractions, effects on amplitude, tension, and frequency	Argentieri and Sheldon, 2006; Rode et al., 2010
	Human	20 mM KCl-induced phasic	Inhibition of tonic contraction	Svalo et al., 2015
	Guinea pig	EFS-induced	Inhibition of phasic contractions, effects on amplitude, muscle force, and duration	Afeli et al., 2013
	Rat	EFS-induced	Inhibition of EFS contractions, effects on amplitude and tension	Rode et al., 2010
	Pig	EFS-induced	Inhibition of EFS contractions (amplitude)	Svalo et al., 2013

Flupirtine (K_V_7.2–K_V_7.5 channel activator)	Guinea pig	Spontaneous phasic	Inhibition of phasic contractions, effects on amplitude and muscle force but not on frequency; also reduced muscle tone	Anderson et al., 2013
	Human	Bethanechol-induced tonic	Inhibition of tonic contraction	Bientinesi et al., 2017

Meclofenamic acid (K_V_7.2/K_V_7.3 channel activator)	Guinea pig	Spontaneous phasic	Inhibition of phasic contractions, effects on amplitude and muscle force but not on frequency; also reduced muscle tone	Anderson et al., 2013

ICA-069673 (K_V_7.2/K_V_7.3 channel activator)	Guinea pig	Spontaneous phasic	Inhibition of phasic contractions effects on amplitude, muscle force, frequency, and duration	Provence et al., 2015
	Guinea pig	Carbachol-induced phasic	Inhibition of phasic contractions, effects on amplitude, muscle force, duration, and frequency	Provence et al., 2015
	Guinea pig	20 mM KCl-induced phasic	Inhibition of phasic contractions, effects on amplitude, muscle force, duration, and frequency	Provence et al., 2015
	Guinea pig	EFS-induced	Inhibition of EFS contractions, effects on amplitude and muscle force	Provence et al., 2015

ML213 (K_V_7.2, K_V_7.2/K_V_7.3, and	Guinea pig	Spontaneous phasic	Inhibition of contractions, effects on amplitude, muscle force, frequency, and duration	Provence et al., 2018
K_V_7.4 channel activator)	Guinea pig	Carbachol-induced phasic	Inhibition of contractions, effects on amplitude, muscle force, duration, and frequency	Provence et al., 2018
	Pig	Carbachol-induced phasic	Inhibition of tonic contractions	Svalo et al., 2013
	Human	Carbachol-induced phasic	Inhibition of tonic contractions	Svalo et al., 2015
	Guinea pig	20 mM KCl-induced phasic	Inhibition of phasic contractions, effects on amplitude, muscle force, duration, and frequency	Provence et al., 2018
	Human	20 mM KCl-induced phasic	No effect on tonic contraction	Svalo et al., 2015
	Guinea pig	EFS-induced	Inhibition of EFS contraction, effects on amplitude and muscle force	Provence et al., 2018

ML277	Human	Carbachol-induced	Inhibition of tonic contractions	Svalo et al., 2015
(K_V_7.1 channel activator)	Human	20 mM KCl-induced	No effect on tonic contraction	Svalo et al., 2015
	Human	40 mM KCl-induced	Inhibition of tonic contraction	Svalo et al., 2015

**TABLE 2:** Effects of K_V_7 channel inhibitors on DSM contractions in various animal species and humans.

**Inhibitor**	**Species**	**Experimental DSM Contraction Protocol**	**Observation on DSM contractility**	**References**
XE991 (K_V_7.1–K_V_7.5 channel blocker)	Guinea pig	Spontaneous phasic	Enhancement of phasic contractions, effects on amplitude and muscle force but weak/unchanged on frequency and duration; also increase or no change in muscle tone	Afeli et al., 2013; Anderson et al., 2013
	Rat	Spontaneous phasic	No effect on contractions	Wang et al., 2014
	Rat	Spontaneous phasic	Enhancement of phasic contractions; effects on amplitude and tension	Rode et al., 2010
	Guinea pig	EFS-induced	Enhancement of EFS contractions, effects on amplitude and muscle force but weak/unchanged on duration; also increase in muscle tone	Afeli et al., 2013
	Rat	20 mM KCl-induced	Enhancement of phasic contractions, effects on amplitude, tension, and frequency	Rode et al., 2010
	Pig	20 mM KCl-induced	Enhancement of phasic contractions, effects on amplitude and tension	Svalo et al., 2013
	Rat	Carbachol-induced	No/weak effects on phasic contractions	Rode et al., 2010
	Pig	Carbachol-induced	No effect on phasic contractions	Svalo et al., 2013
	Pig	EFS-induced	No effect on EFS contractions	Svalo et al., 2013
	Human	Spontaneous tonic activity (lacking phasic contractions)	Enhancement of tonic contraction	Bientinesi et al., 2017
	Human	20 mM K^+^-induced	Enhancement of tonic contraction	Svalo et al., 2015
	Human	40 mM K^+^-induced	No effect on tonic contraction	Svalo et al., 2015
	Human	Carbachol-induced	No effect on tonic contraction	Svalo et al., 2015

Linopirdine (K_V_7.1–K_V_7.5 channel blocker)	Guinea pig	Spontaneous phasic	Enhancement of phasic contractions, effects on amplitude and muscle force but weak/unchanged on frequency and duration; also increase or no change in muscle tone	Afeli et al., 2013; Anderson et al., 2013
	Guinea pig	EFS-induced	Enhancement of EFS contractions, effects on amplitude and muscle force but weak/unchanged on duration; also increase in muscle tone	Afeli et al., 2013

Chromanol 293B (K_V_7.1 channel blocker)	Guinea pig	Spontaneous phasic	Enhancement of phasic contractions, effects on amplitude and muscle force but weak/unchanged on frequency also no effect on muscle tone	Anderson et al., 2013
	Pig	20 mM KCl-induced	No effect on phasic contractions	Svalo et al., 2013
	Pig	EFS-induced	No effect on EFS contractions	Svalo et al., 2013
	Pig	Carbachol-induced	No effect on phasic contractions	Svalo et al., 2013

The rat is the next most common animal species in which DSM K_V_7 channels have been studied, but only three publications are currently available (Argentieri and Sheldon, 2006; Rode et al., 2010; Wang et al., 2014) and a single report on pig DSM (Svalo et al., 2013). In rat DSM, retigabine and ML213 effectively inhibited DSM spontaneous phasic, 20 mM KCl-induced phasic, or carbachol-potentiated phasic contractions (Argentieri and Sheldon, 2006; Rode et al., 2010; Wang et al., 2014). The elevation of extracellular K^+^, which results in a reduction of the driving force for K^+^, caused attenuation in retigabine-induced relaxation (Argentieri and Sheldon, 2006). This finding indicates that retigabine effects on DSM involve activation of K^+^ channels. In contrast, the inhibitor XE991 displayed either no effect or a strong potentiation of DSM contractility (Rode et al., 2010; Wang et al., 2014). The reason for the differential experimental outcomes for XE991 remains to be resolved. In pig DSM, retigabine inhibited both EFS- and carbachol-induced contractions only in the absence but not in the presence of XE991. XE991 alone enhanced 20 mM KCl-induced but not EFS-induced contractions; chromanol 293B was ineffective in altering either of these two types of stimulated contractions, indicating a negligible role of K_V_7.1 channels in pig DSM (Svalo et al., 2013). In mouse DSM, the effects of retigabine and XE991 on isolated strip contractility have only been described, so far, under the condition of highly elevated extracellular K^+^ (60 mM) and the resultant non-physiological tonic contraction (Tykocki et al., 2019). Since responses to K_V_7 channel modulators have not yet been examined under conditions of physiological extracellular K^+^ concentration (i.e., ∼5 mM K^+^), the role of K_V_7 channels in mouse DSM strip contractility remains to be determined. The limited number of publications on rat, pig, and mouse DSM seeking to determine pharmacological modulation of K_V_7 channels provide inconsistent findings and this scientific controversy needs clarification. Given that these initial studies found substantial pharmacological modulatory effects on contractility with K_V_7 channels in these animal models, additional studies of the effects of new and improved subtype-specific K_V_7 channel modulators are highly warranted.

#### Initial Studies on Human DSM Contractility

The gap in the understanding of DSM K_V_7 channels lies in the lack of systematic studies on human DSM contractility; such studies are already ongoing in our laboratory in collaboration with clinical urologists. Currently, there are only two publications with very limited data available on this topic (Svalo et al., 2015; Bientinesi et al., 2017). In an earlier study, retigabine inhibited DSM contractility that was enhanced by carbachol and K^+^ (20 or 40 mM) (Svalo et al., 2015). ML213 also inhibited carbachol-potentiated but not KCl-enhanced DSM contractions (Svalo et al., 2015). The K_V_7.1 channel activator ML277 displayed inhibitory effects on carbachol- and 20 mM KCl-induced contractions, but not on 40 mM KCl (Svalo et al., 2015). XE991 could only increase contractions potentiated by 20 mM K^+^, but not by carbachol or 40 mM K^+^ (Svalo et al., 2015). The specific experimental conditions employed, hence, influenced the outcome of pharmacological experiments. The second study on human DSM showed that DSM strips exhibiting quiescence and lacking phasic contractions responded to increasing concentrations of XE991 with the development of step-wise enhanced tonic contractions (Bientinesi et al., 2017). While application of XE991 caused an appearance of DSM phasic contractions, they were very low in amplitude and therefore could not be reliably analyzed (Bientinesi et al., 2017). Further, retigabine and flupirtine attenuated muscarinic agonist (bethanechol)-potentiated DSM tonic contractions (phasic contractions were minimal under the stimulated condition) in a concentration-dependent manner (Bientinesi et al., 2017). The effects of flupirtine and retigabine were reduced in the presence of XE991 but not affected by either the Na_V_ channel inhibitor tetrodotoxin or the N-type Ca_V_ blocker ω-conotoxin GVIA. The lack of efficacy of tetrodotoxin as well as ω-conotoxin GVIA on retigabine- and flupirtine-induced DSM relaxations suggested that K_V_7 channels expressed on DSM cells mediated the inhibitory effects of these two activators (Bientinesi et al., 2017). These two reports — while valuable, interesting, and providing some initial evidence for a role of K_V_7 channels in regulating human DSM contractility — have multiple experimental limitations (Svalo et al., 2015; Bientinesi et al., 2017). The stimulated (e.g., carbachol and high KCl) and non-stimulated DSM contraction patterns recorded had all very low amplitudes, and the only experimental variable reliably quantified was average muscle tone. Other DSM phasic contraction parameters, especially amplitude, muscle force, duration, and frequency, were not quantified. Since these characteristics can be measured only for robust contractions (as previously reported by our group and others) on human DSM (Sibley, 1984; Darblade et al., 2006; Hristov et al., 2011, Hristov et al., 2012a, Hristov et al., 2012c, Hristov et al., 2013, Hristov et al., 2016, Hristov et al., 2017; Oger et al., 2011; Afeli et al., 2012; Soder et al., 2013; Xin et al., 2016), additional studies conducted under physiologically relevant experimental conditions are needed. The second limitation involves the absence of spontaneous phasic contractions under non-stimulated conditions (e.g., without elevated K^+^ or cholinergic agonists). These DSM spontaneous contractions are attributed to the inherent intrinsic contractility of DSM cells; also, when excessive, they drive the development of DO (Brading et al., 1986; Turner and Brading, 1997; Andersson and Arner, 2004). The third limitation lies in the current lack of knowledge of how K_V_7 channel modulators alter bladder contractility under pathophysiological conditions. Future and ongoing systematic studies on human DSM contractility — including those underway in our laboratory — that will overcome these limitations are needed to comprehensively validate K_V_7 channel subtypes as critical regulators of human DSM contractility, and to identify them as potential therapeutic targets for urinary bladder dysfunction.

#### Pharmacological Effects of K_V_7 Channel Modulators on DSM Intracellular Ca^2+^ Concentration

In DSM as in other types of smooth muscle preparations, intracellular Ca^2+^ regulates contractility (Andersson and Arner, 2004; Andersson and Wein, 2004). Thus, it is important to elucidate how K_V_7 channel modulators affect intracellular Ca^2+^ concentrations in DSM cells. Currently, only studies on guinea pig DSM are available in the literature (Anderson et al., 2013; Provence et al., 2015, 2018). DSM muscle bundles displayed either coordinated whole bundle Ca^2+^ flashes or spontaneous Ca^2+^ transients and smaller localized Ca^2+^ events in individual DSM cells, detected using the Ca^2+^ fluorescence indicator Fluo-4AM (Anderson et al., 2013). The K_V_7 channel blocker XE991 increased all three types of Ca^2+^ events in DSM cells (bundle Ca^2+^ flashes, individual whole-cell Ca^2+^ oscillations, and localized Ca^2+^ events) (Anderson et al., 2013). Although that particular study (Anderson et al., 2013) did not examine the effects of a K_V_7 channel activator, two more recent studies by our group did (Provence et al., 2015, 2018). We found that intracellular Ca^2+^ concentration in DSM tissue and cells, imaged by Fura-2AM, was decreased following the application of two subtype-preferential K_V_7 channel activators, ICA-069673 (Provence et al., 2015) and ML213 (Provence et al., 2018). Further, the study with the channel blocker ML213 showed that pretreatment of DSM with nifedipine, an L-type Ca_V_ channel blocker, prevented the decrease in intracellular Ca^2+^ concentration induced by the K_V_7 channel activator (Provence et al., 2018). This finding provided experimental support that the K_V_7 channel activator-induced decrease in intracellular Ca^2+^ concentrations mechanistically involves L-type Ca_V_ channels. Since the studies examining the effects of K_V_7 channel modulators on intracellular Ca^2+^ concentration are limited only to the guinea pig and to a very few compounds tested, additional investigations are needed. These critical follow-up studies on DSM that are crucial in humans should also be examined in animals models including rat, mouse, and pig testing novel subtype-specific K_V_7 channel modulators under highly optimized experimental imaging conditions (such as newer generation Ca^2+^ dyes, genetically encoded Ca^2+^ indicators selectively expressed in DSM cells, and high resolution, fast speed, whole-tissue microscopy). Our laboratory has already initiated systematic studies in this context.

## Discussion and Closing Remarks

In this comprehensive review, we have summarized the current knowledge of DSM K_V_7 channels, highlighting expression profiles (mRNA and protein) in DSM whole-tissue and single cells and pharmacological effects of K_V_7 channel modulators on DSM excitability (whole-cell patch-clamp and sharp microelectrode electrophysiology), intracellular Ca^2+^ concentrations (muscle bundle/tissue and DSM cells), and DSM contractility examined in various species (guinea pig, rat, mouse, pig, and human). Since humans are the target for therapeutic intervention, determining how K_V_7 channels regulate human urinary bladder function at the cellular, tissue, organ, and whole-body levels is essential. However, our present understanding is rudimentary and limited to only a few studies. Although the initial clinical finding from epilepsy clinical trials identified urinary bladder retention as a retigabine use-associated side effect that has been further supported by animal *in vivo* and *in vitro* studies, the concept that K_V_7 channels provide a potential novel therapeutic target for overactive bladder remains to be adequately validated. Additional systematic studies on human DSM are needed to fill current critical gaps in knowledge to determine how K_V_7 channels regulate urinary bladder function under normal and pathophysiological conditions. Our laboratory has already initiated systematic investigations in this area in collaboration with clinical urologists from multiple clinical settings in the US. These studies will validate the K_V_7 channel as a viable therapeutic target for urinary bladder dysfunction.

Among the animal models examined, most studies of DSM K_V_7 channels, until now, have been conducted primarily on guinea pigs, which have revealed expression of all K_V_7 channel subtypes (K_V_7.1–K_V_7.5) and functional roles for K_V_7 channel subtypes in determining DSM cell excitability, intracellular Ca^2+^ concentration, and tissue contractility. Guinea pig DSM, however, displays a differential K_V_7 channel subtype expression profile compared to that of humans and rats. Further, *in vivo* urinary bladder functional assessments with K_V_7 channel modulators in guinea pigs are lacking. The translational usefulness of the mouse model for DSM K_V_7 channel studies has been questioned (Tykocki et al., 2019).

Therefore, the rat appears to be the best animal model for future studies of DSM K_V_7 channels given the subtype expression similarity to human (whole-tissue level), the already demonstrated *in vivo* efficacy of K_V_7 channel modulators on urinary bladder function, and initial DSM excitability and contractility outcomes. To fully validate the rat model, additional studies are needed on single DSM cells and tissues illustrating expression and functional roles of K_V_7 channels, including supportive findings based on dysfunction in smooth muscle-specific K_V_7 channel animal knock-out models.

While in this review we have focused on DSM, with an emphasis on K_V_7 channel expression and its functional roles, K_V_7 channels expressed on urinary bladder innervating neurons and fibers, in the spinal cord, and the brain, as well as non-DSM cells (e.g., interstitial cells) in the bladder can affect overall urinary bladder function. Indeed, some limited experimental evidence has been provided for K_V_7 channels expressed in interstitial cells and dorsal root ganglia/sensory afferents (Anderson et al., 2009; Tykocki et al., 2019). These additional cell types provide other opportunities for future research endeavors on K_V_7 channels in the urinary bladder.

In conclusion, the currently available experimental evidence strongly supports the functional expression and regulatory roles of K_V_7 channels in DSM. The reported findings, however, are based on limited studies in humans and animal models, as summarized here. To advance our understanding of K_V_7 channel subtypes in DSM and urinary bladder, additional dedicated research efforts on human DSM tissues and cells (obtained from patient donors exhibiting healthy/control and pathological urinary bladder phenotypes) as well as a translationally relevant animal model, such as the rat, are urgently needed. The already initiated systematic research investigations in collaboration with clinical urologists at our Urology Research Center, University of Tennessee, Memphis will reveal how DSM K_V_7 channels impact urinary bladder function and whether they can be targeted for management of urinary bladder diseases.

## Author Contributions

JM prepared the initial draft, edited the content, and approved the final version. GP edited the initial draft and content, and approved the final version. Both the authors contributed to the article and approved the submitted version.

## Conflict of Interest

The authors declare that the research was conducted in the absence of any commercial or financial relationships that could be construed as a potential conflict of interest.

## Abbreviations

BPH: benign prostatic hyperplasia
Ca_V_: L-type voltage-gated Ca^2+^ (channel)
DO: detrusor overactivity
DSM: detrusor smooth muscle
EFS: electrical field stimulation
ICA-069673: *N*-(2-Chloro-5-pyrimidinyl)-3,4-difluorobenzamide
K_V_7: voltage-gated potassium type 7 (channel)
KCNE: K^+^ voltage-gated channel subfamily E (member)
ML213: *N*-(2,4,6-Trimethylphenyl)-bicyclo[2.2.1]heptane-2-carboxamide
ML277: (2R)-*N*-[4-(4-Methoxyphenyl)-2-thiazolyl]-1-[(4-methylphenyl)sulfonyl]-2-piperidinecarboxamide
MMSC: muscularis mucosae smooth muscle
qRT-PCR: quantitative reverse transcription polymerase chain reaction
RBC: rhythmic bladder contraction
SMC: smooth muscle cell
TC: transient contraction
XE991: 10,10-bis(4-Pyridinylmethyl)-9(10H)-anthracenone dihydrochloride.
